# Assessment of Associations Between Sociodemographic and Analysis of Risk Factors for Oral Infectious Pathology in Patients Scheduled for Total Hip and Knee Arthroplasty

**DOI:** 10.3390/clinpract15120220

**Published:** 2025-11-24

**Authors:** Dana Nicoleta Mihai, Paul Dan Sîrbu, Liliana Savin, Norin Forna, Claudiu Topoliceanu, Cristina Dascălu, Norina Consuela Forna

**Affiliations:** Grigore T. Popa University of Medicine and Pharmacy Iasi, Universitatii Street 16, 700115 Iasi, Romania; mihaidana0232@yahoo.com (D.N.M.); paul.sirbu@umfiasi.ro (P.D.S.); liliana.savin@umfiasi.ro (L.S.); claudiu.topoliceanu@umfiasi.ro (C.T.); cristina.dascalu@umfiasi.ro (C.D.); norina.forna@umfiasi.ro (N.C.F.)

**Keywords:** total arthroplasty, oral infectious pathology, oral foci of infection, risk factors

## Abstract

The aim of this study was to evaluate the factors associated with the occurrence of oral infection sources in patients scheduled for total hip or knee arthroplasty, with the purpose of establishing standardized preoperative dental triage criteria. **Materials and Methods:** A retrospective research was conducted on a study group of 89 patients diagnosed with hip osteoarthritis and knee osteoarthritis at the Clinical Rehabilitation Hospital (Iasi, Romania). Patients were divided according to the status of their oral cavity: study group (*n* = 51)—patients with diagnosed oral infection sites (oral foci of infection); control group (*n* = 38)—patients without oral foci of infection. The statistical analysis included a univariate stage followed by a multivariate binary logistic regression to identify demographic and clinical factors associated with the presence of oral foci of infection. **Results:** The strongest predictor of the presence of oral foci of infection was and Oral Hygiene Index (OHI) scorer of 2, which increased the risk 14.583-fold, followed by being aged between 50 and 65 years (OR = 4.038), tooth brushing once a day or less (OR = 3.488), and male sex (OR = 3.433). An OHI score of 2 raises the probability of oral infectious pathology to 30.3%, which increases to 85.1% when combined with being aged between 50 and 65 years. **Conclusions:** The risk factors for the presence or oral foci of infection in patients scheduled for total knee or hip arthroplasty support the inclusion of the preoperative assessment and management of these factors in order to reduce the risk of the postoperative periprosthetic joint infections.

## 1. Introduction

Total joint arthroplasty of the hip (THA—Total Hip Arthroplasty) and knee (TKA—Total Knee Arthroplasty) are reliable procedures for restoring joint function in patients with knee or hip osteoarthrosis [1]. Periprosthetic joint infection (PJI) has been reported as major factor of failure in 1–2% of total joint arthroplasties [2], with an incidence between 0.5% and 1% in total hip arthroplasty [3,4] and 0.5% and 2% in total knee arthroplasty [4,5]. A research group reported a cumulative incidence in TKA of 6.1% at 15 years of follow-up, with a 2.0% revision rate due to periprosthetic joint infections (PJIs) [6]. A research group showed that, compared with aseptic revision TKA, revision procedures for PJI increase the risk of death by between 3.25 times [7] and 3.52 times [8] when compared with aseptic revision of TKA. Revision of TKA due to PJI brings a higher risk of short-term morbidity and mortality and an increase in healthcare costs, disability, and reinfection [9]. A research group reported significantly higher mortality rates among patients who developed periprosthetic joint infection compared with those who underwent aseptic revision arthroplasty, both in the short and long term—3.7% vs. 0.8% at 90 days, 10.6% vs. 2.0% at 1 year, 13.6% vs. 3.9% at 2 years, and 25.9% vs. 12.9% at 5 years [10]. To avoid these consequences, the prevention of PJI requires preoperative patient assessment, medical optimization, and antibiotic prophylaxis [11]. Patients scheduled for THA and TKA must undergo preoperative screening, and postoperative sepsis prophylaxis which include the identification and treatment of oral infections as well as infectious sources localized in skin, respiratory, gastrointestinal, and urogenital systems [12].

In this regard, international health authorities and organizations underline the importance of oral health as an essential part of general health, promoting the integration of dental care into overall medical practice [13]. During the last two decades, the importance of oral health in preoperative care has become increasingly acknowledged among patients undergoing joint arthroplasty, including THA and TKA procedures. Unclear responsibilities, low awareness, as well as insufficient prioritization have been identified as barriers to effective integration of oral healthcare into basic health services [14]. Also, data regarding the role of oral pathogens in the development of PJI remain inconsistent, with reported incidence ranging from 0.03–0.04% [15,16] to 6–13% [17,18]. At the level of low-dependency facilities, important aspects must be addressed such as leadership involvement, supportive attitudes of caregivers, and patient responsibility. This shift reflects a broader understanding of the link between oral and general health, as well as the specific surgical risks involved.

Considering the burden of oral infectious pathology in patients scheduled for hip and knee arthroplasty, as reported in the literature [19,20,21], we believe that comprehensive dental examination and oral treatment planning should be an integral part of preparation for all patients scheduled for knee or hip total arthroplasty. In daily practice, however, this is not a common approach due to the significant implications for patients in terms of costs and inconvenience. This highlights the necessity of at least a limited medical screening performed by the orthopedic surgeon to identify relevant risk factors and, if necessary, to refer the patient for an appropriate dental assessment [22,23].

The aim of this study was to evaluate the factors associated with the occurrence of oral infection foci in patients scheduled for total hip or knee arthroplasty, with the purpose of establishing standardized preoperative dental triage criteria.

## 2. Materials and Methods

### 2.1. Study Design

This retrospective research was conducted on a study group of 89 patients diagnosed with hip osteoarthritis and knee osteoarthritis, scheduled for hip or knee arthroplasty at the Clinical Rehabilitation Hospital (Iasi, Romania).

The research was carried out in accordance with the ethical principles of the Declaration of Helsinki and received approval from the Research Ethics Committee of “Grigore T. Popa” University of Medicine and Pharmacy Iași (approval number: 614; approval date: 22 June 2025). The participation consent form was distributed to and signed by all participants. According to the presence or absence of infectious oral pathology the patients were divided in two groups. The study group (OFI—oral foci of infection) consisted of 51 patients with oral infection sites. The mean age in this group was 67.51 ± 5.78 years. Inclusion criteria were the presence of oral infectious pathology in the oral cavity, age over 50 years, eligibility for hip or knee osteoarthritis surgery, and availability for a complete dental examination. Exclusion criteria included revision arthroplasty or previous joint prostheses, systemic infections at the time of evaluation, total edentulism, incomplete medical records, and unavailability for dental examination. The control group (Non-OFI—non-oral foci of infection) included 38 patients without oral infectious sites, with a mean age of 69.32 ± 5.90 years. Inclusion criteria were age over 50 years, eligibility for hip or knee osteoarthritis surgery, and availability for a complete dental examination. Exclusion criteria were the presence of infectious pathology in the oral cavity, revision arthroplasty or previous joint prostheses, systemic infections at the time of evaluation, total edentulism, incomplete medical records, and unavailability for dental examination.

The common features of the selected patients are shown in Table 1. Among them, 29 were men (32.6%) and 60 were women (67.4%); 61 patients came from urban areas (68.5%) and 28 from rural areas (31.5%). With respect to age distribution, 28 patients were between 50 and 65 years old (31.5%), while 61 were over 65 years old (68.5%). Twelve participants were smokers (13.5%), whereas 77 were non-smokers (86.5%). Chronic alcohol consumption was reported by 16 patients (18.0%). More than half of the subjects had at least one systemic pathology (50 patients; 56.2%), including diabetes mellitus in 38 cases (42.7%) and rheumatoid arthritis in 15 cases (16.9%). Obesity was present in 69 individuals (77.5%), and eight patients (9.0%) were receiving immunosuppressive treatment. The orthopedic diagnoses were almost equally distributed, with 45 patients suffering from knee osteoarthritis (50.6%) and 44 from hip osteoarthritis (49.4%). Only 32 patients (36.0%) reported undergoing annual dental check-ups. Regarding tooth brushing frequency, 41 patients (46.1%) reported brushing their teeth once a day, 43 (48.3%) reported brushing their teeth several times a day, and 5 (5.6%) reported brushing their teeth once every few days. The Oral Hygiene Index (OHI) indicated good oral hygiene (score 0) in 20 patients (22.5%), moderate oral hygiene (score 1) in 27 patients (30.3%), and poor oral hygiene (score 2) in 42 patients (47.2%). In the last three months, 8 patients (9.0%) reported a dental infection, 12 (13.5%) underwent a tooth extraction, and 8 (9.0%) received root canal treatment (Table 1). Features of the selected subjects are shown in Table 1.

Criteria definition:

- Smoker: individuals who reported smoking at least one cigarette per day for a minimum period of six months prior to examination [24].
- Chronic alcohol consumption: individuals who consumed alcoholic beverages on a regular basis (≥3 times per week) for more than one year, regardless of beverage type [25].
- Obesity: body mass index (BMI) ≥ 30 kg/m^2^, according to the World Health Organization (WHO) classification [26].

### 2.2. Diagnosis of Orthopedic Pathology 

An orthopedic physician diagnosed hip or knee osteoarthritis based on established clinical and radiological criteria. Clinically, the diagnosis was confirmed by a history of chronic joint pain, restricted mobility, and the presence of crepitus during joint movement. Radiological confirmation was performed according to the Kellgren–Lawrence classification, with a score ≥ 2 considered diagnostic. All eligible patients were scheduled for THA or TKA for advanced forms of symptomatic osteoarthritis refractory to conservative treatments [27].

### 2.3. Diagnosis of Oral Infectious Pathology (Oral Foci of Infection)

A dental specialist performed a standardized clinical and paraclinical intraoral examination on all patients. Clinical oral examination consisted of visual and tactile examination performed under adequate lighting and aseptic conditions. OHI was calculated by a formula combining the debris index (DI) and calculus index (CI), which assess the amounts of debris or calculus on the dental buccal and lingual surfaces. Both the DI and CI are quantified by a 4-point scale (0, 1, 2, 3) [28]. The Oral Hygiene Index (OHI) was determined by combining the debris index (DI) and the calculus index (CI), evaluated on six representative teeth. Each index was scored on a 4-point scale (0–3) according to the amount of debris or calculus observed on tooth surfaces. To obtain a simplified categorical OHI score for risk analysis, the following formula and classification were applied: OHI = (DI + CI)/2. The mean OHI value was then categorized as follows: OHI = 0 (mean < 1)—good oral hygiene; OHI = 1 (mean ≥ 1 and <2)—moderate oral hygiene; OHI = 2 (mean ≥ 2)—poor oral hygiene This categorization ensured that all participants were classified into one of three distinct hygiene levels (0, 1, or 2).

Clinical oral examination was carried out with mouth mirror, explorer, and tweezers (Aesculap AG, Tuttlingen, Germany). The examinations of the periodontal areas were performed using a manual periodontal probe (Click-Probe^®^, Kerr, Bioggio, Switzerland), calibrated in millimeters for measuring pocket depth. Additional radiographic imaging (panoramic radiographs and periapical radiography) was performed for a complete diagnosis of periapical lesions and periodontal disease. Radiographic examinations were performed with a digital dental X-ray unit (Planmeca ProX^®^, Planmeca Oy, Helsinki, Finland). Marginal bone loss (MBL) was assessed using a millimeter-graded ruler under ×2 magnification (Eschenbach Mobilux LED Magnifier, Eschenbach Optik, Nuremberg, Germany). Sources of oral foci of infection were defined as follows:

- Chronic periapical lesions (CPLs) were diagnosed at the tooth apex by detection on radiographic images of a curved or circular shape and a radiolucent internal structure, consistent with a chronic inflammatory process [29].
- Endo-perio lesions (EPLs) were diagnosed by detection of clinical signs (deep periodontal pockets extending to or near the apex, presence of pus drainage, increased mobility) and radiographic image of vertical bone loss along the root and/or periapical radiolucency [30]. The major diagnostic criteria of EPLs were as follows: (1) periodontal probing depth (PPD) ≥ 4 mm, (2) clinical attachment loss (CAL) ≥ 3 mm, and (3) patients with pulp symptoms, such as spontaneous pain history or negative or altered pulp vitality tests [31].
- Periodontal disease. Diagnosis of the stage and progression of periodontal pathology considered anamnestic data as well as clinical and imagistic data collected before scheduling a patient for arthroplasty. Periodontal pocket depth (PPD) were measured at 4 sites for all teeth using a manual periodontal probe (Click-Probe®, Kerr, Bioggio, Switzerland). Active PPDs ≥ 4 mm were considered oral infectious foci. Marginal bone loss (MBL) was measured on orthopantomograms with a millimetergraded ruler under ×2 magnification using a magnifying viewer, from the cemento-enamel junction (CEJ) to the alveolar bone crest, at the mesial and distal proximal tooth sites. Values were rounded off to the nearest 0.1 mm. The stage and grading of periodontal disease were assessed according to the 2017 World Workshop on the Classification of Periodontal and Periimplant Diseases, as follows: staging (I, II, III, and IV) and grading (A, B, and C) [32,33]. Advanced periodontal disease in stages III or IV (grades B/C) was included in the category of oral infectious foci.
- Root remnants—untreated or previously treated residual root fragments as well as fractured roots after extraction were considered oral sources of infection.
- Fixed prosthetic restorations with improper marginal adaptation and active periodontal pockets (clinical and radiographic evaluation) were considered oral sources of infection [34].

### 2.4. Statistical Analysis

To identify the factors associated with the presence of oral foci of infection, we performed a univariate analysis, followed by a multivariate analysis using a binary logistic regression model. In the univariate analysis, we identified associations between patients’ demographic and clinical characteristics and the presence of oral foci of infection using Pearson’s Chi-square test. If statistically significant associations were found, we calculated odds ratios (ORs), relative risk (RR) and the corresponding 95% confidence intervals. In the multivariate analysis stage, we applied a binary logistic regression model, the Forward LR method, to investigate the combined influence of the previously identified risk factors on the presence of oral infectious foci. The analysis was carried out in three successive steps, with the gradual inclusion of independent variables considered clinically relevant. The dependent variable (oral infectious foci) was dichotomous (0 = absence; 1 = presence). The reliability of the model was evaluated using the Omnibus Tests of Model Coefficients and the Hosmer–Lemeshow test, while its accuracy was assessed by calculating the Nagelkerke R-Squared coefficient. For each independent variable, the regression coefficient (B), standard error (S.E.), Wald statistic, significance level (p), and odds ratio (Exp(B)) with 95% confidence interval were calculated. Statistical significance was set at *p* < 0.05, while values of *p* < 0.01 were considered highly statistically significant. The analysis was performed in SPSS software, version 29.0.

## 3. Results

Table 2 shows the prevalence rates of various categories of oral foci of infection in patients scheduled for knee or hip arthroplasty. Prevalence rates were as follows: chronic periapical lesions—23.5%; endo-periodontal—41.2%; root remnants—84.3%; advanced periodontal disease (at least one active PPD ≥ 4 mm); and fixed prosthetic with improper marginal fitting—64.7%.

The comparative results on socio-demographic parameters, systemic status, oral hygiene, and dental history in patients scheduled for total hip or knee arthroplasty is shown in Table 3. Univariate analysis showed that men had significantly more oral foci of infection than women (44.7% vs. 19.0%; *p* = 0.010). Place of residence did not significantly influence the results (urban 63.8% vs. 73.8%; *p* = 0.311). Age 50–65 years was associated with more than a fourfold higher risk compared with the >65 group (44.7% vs. 16.7%; *p* = 0.004). Smoking doubled the relative risk of oral foci of infection (25.5% vs. 0%; *p* < 0.001), while chronic alcohol consumption had no significant impact (*p* = 0.804). Systemic pathologies overall, diabetes mellitus, and obesity did not reach the threshold of significance, but rheumatoid arthritis was inversely associated with the presence of oral foci of infection (8.5% vs. 26.2%; *p* = 0.026). Immunosuppressive therapy doubled the relative risk (17.0% vs. 0%; *p* = 0.006). The absence of annual dental check-ups showed only a trend toward significance (*p* = 0.070). Tooth brushing once a day or less substantially increased the risk (66.0% vs. 35.7%; *p* = 0.004), while tooth brushing several times per day was associated with a reduced risk (34.0% vs. 64.3%; *p* = 0.005). An OHI score = 2 was the strongest univariate predictor: 74.5% of cases vs. 16.7% (*p* < 0.001). Moreover, any OHI ≥ 1 was significantly associated with infection (*p* < 0.001). Recent dental events increased the risk: dental infection, root canal treatment, or other dental procedures in the last 3 months approximately doubled the relative risk (*p* ≤ 0.006). Recent extractions were not significantly associated (*p* = 0.301). Overall, the significant risk factors in univariate analysis were as follows: male sex, age group 50–65 years, smoking, immunosuppressive therapy, tooth brushing once a day or less, OHI 2, and recent history of oral infection or endodontic treatment (Table 3).

According to the univariate analysis, several factors were significantly associated with the presence of oral infection foci in patients scheduled for total hip or knee arthroplasty.

Male patients had a 3.433-fold higher risk compared to females; those in the 50–65 age group had a 4.038-fold higher risk than patients over 65 years. Smoking was associated with a relative risk (RR) of 2.200. The administration of immunosuppressive drug therapy was linked to a 2.077-fold increased risk. The habit of tooth brushing once every few days was associated with a relative risk of 2.000, whereas tooth brushing once a day or less increased the risk by 3.488 times. The strongest predictor identified was an OHI score = 2, which increased the risk of oral foci of infection by 14.583 times. The presence of oral infection sites within the last 3 months was associated with a relative risk of 2.077, while recent endodontic treatments (within the last 3 months) showed the same risk (RR = 2.077). Other dental treatments performed in the same period increased the risk 2.400 times. These predictors highlight the profiles of patients at increased risk of presenting oral foci of infection prior to total knee or hip arthroplasty (Table 4).

The results of the multivariate logistic regression analysis indicate the existence of four statistically significant risk factors for the presence of oral foci of infection in patients scheduled for total hip or knee arthroplasty.

The risk factors included in the model were patient sex (male), age group (50–65 years), tooth brushing (once daily or less), and OHI score (=2).

The first risk factor identified was OHI 2 (poor oral hygiene). This variable was associated with a 16.314-fold increase in the risk of oral foci of infection compared to patients with an OHI score < 2. The next risk factor, added at step 2 of the model, was the age group 50–65 years. This variable was also associated with a high risk, with a 13.168-fold higher probability of presenting oral foci of infection compared to patients over 65 years. The third risk factor was male sex, associated with a 12.050-fold increased risk, also highly statistically significant. The last risk factor introduced into the model was poor oral hygiene behavior, specifically tooth brushing once a day or less. This habit increased the risk of presenting oral foci of infection by 7.951 times compared to brushing twice daily or more (Table 4).

## 4. Discussion

The prevalence of infectious pathology in the oral cavity is considerable, being represented by advanced periodontal disease [35,36], chronic periapical lesions [37,38,39], endo-perio lesions [40,41], as well as prosthetic restorations with improper marginal adaptation and active periodontal pockets [42,43,44], all of which constitute major sources of oral infection.

The starting point of this study was the lack of epidemiological and clinical data related to oral infectious pathology in elderly patients seeking surgical services such as total hip or knee arthroplasty. Understanding the complexity of oral pathology in this category of patients is essential for providing quality medical services, both in terms of diagnosis and treatment. Moreover, the correct identification of oral diseases in elderly patients requires a careful evaluation of the general health status, including the underlying disease and associated comorbidities. To adequately respond to these needs, it is important that the approach to elderly patients with oral infectious pathology be transdisciplinary and interdisciplinary [45,46,47,48].

The relationship between OHI indices, oral inflammation, and periprosthetic joint infection is mediated by the oral foci in the oral cavity. Bacteria from various categories of oral foci are able to enter into the bloodstream, especially during mastication or dental treatments. This bacteremia may trigger immune activation and inflammatory cascades, which in vulnerable patients—such as those scheduled for arthroplasty—can potentiate systemic inflammatory responses and increase the risk of periprosthetic joint infections.

In a discussion about infectious pathology of oral cavity, various risk factors must be taken in consideration [49,50,51]. Although the direct causal relationship between dental procedures and periprosthetic joint infections is not unanimously accepted, the identification of risk factors for oral infectious pathology within a preventive approach is necessary [52]. Even though not all of these treatments automatically imply an increased risk of postoperative complications, it is clear that an unstable oral cavity, with recent interventions, must be carefully monitored before arthroplasty [53,54,55,56,57,58].

When comparing the analyzed categories, marked differences can be observed in the prevalence of oral foci of infection, depending on certain individual and behavioral characteristics. For example, although place of residence (urban vs. rural) did not significantly influence the results, patients from rural areas had a slightly higher frequency of oral foci of infection (36.2% vs. 26.2%), suggesting possible barriers to accessing dental care services or differences in oral hygiene habits. The results show that nearly half of the patients had not attended a dental consultation for several years. This is an alarming signal, especially for individuals undergoing major interventions such as arthroplasty. Regular dental check-ups are not merely a formality—they play an essential role in the early detection of infections or dental problems which, if left untreated, can impact general health. In our study, all patients scheduled for hip or knee arthroplasty were over 50 years of age, with a large proportion belonging to the group aged over 65.

A research group has highlighted the increased prevalence of oral infectious pathology among hospitalized elderly patients in Romania [59]. These results were recorded in a context in which, in Romania, dental services are provided mainly by dentists working in private practices and clinics. According to available national data, the number of dental consultations and treatments carried out between 2011 and 2020 decreased by approximately 30%, suggesting reduced patient access to dental care providers. Although the National Health Insurance House reimburses part of these services, the budget allocated to oral health remains insufficient to cover the real needs of the population [60]. In the public hospital system, the only unit offering dental treatments is the oral and maxillofacial surgery department, where specialists provide care for maxillofacial conditions.

To better understand the factors contributing to the occurrence of dental infectious foci, especially among patients on the verge of major surgery such as arthroplasty, multivariate analysis becomes an extremely valuable tool. The comparative analysis of patients scheduled for hip or knee arthroplasty revealed significant differences between the investigated subgroups, confirming that certain demographic and behavioral characteristics represent important risk factors for the presence of oral infectious foci. Consequently, men, patients aged between 50 and 65 years, smokers, and individuals with poor oral hygiene (OHI 2) showed an increased prevalence of infectious lesions. Overall, these results confirm the initial research hypothesis stated above, namely that the patient’s profile can significantly influence oral status and the risk associated with potentially invasive prosthetic interventions.

The results of the multivariate analysis provided additional relevant information, identifying four independent risk factors for the occurrence of oral infectious foci: female sex, age over 65 years, tooth brushing twice daily or more, and OHI < 2. Interestingly, the association of all four risk factors led to an almost certain probability (99.8%) of developing an infectious focus, which underlines the cumulative and interactive effect of these seemingly paradoxical factors (particularly frequent tooth brushing and OHI < 2, which would suggest a favorable profile). Among all predictors, an OHI score = 2 was the most influential, its presence alone being sufficient to increase the probability of infection to 30.3%, even without other risk factors. When combined with the second risk factor—age between 50 and 65 years—the risk increased to 85.1%. In our study population, patients aged 50–65 years were significantly more likely to present oral foci of infection compared with those over 65 years (OR = 4.038, *p* = 0.004). This result can be explained by factors related to the patients’ behavior rather than age itself. Patients in the 50–65 group are still professionally active, and may have limited time for regular dental care, while they frequently present with incomplete or neglected dental treatments. Older patients (>65 years) are more likely to be edentulous or have received prior extractions that eliminated potential oral foci. These results confirm that an adequate oral hygiene status is not limited to brushing frequency but also depends on brushing efficiency, as reflected in the OHI score. The absence of all four risk factors identified in the multivariate analysis was associated with an extremely low probability of infection (2.6%). This observation has major clinical implications, as it allows the definition of a low-risk patient profile, in which orthopedic interventions can be performed under safer conditions. Overall, the findings confirm that demographic, behavioral, and clinical factors all contribute to the risk of developing oral foci of infection in patients scheduled for arthroplasty.

The importance of assessing and correcting oral hygiene before major surgical intervention is evident, given the potential risk of periprosthetic infections. Our findings reflect not only the lack of an effective tooth-cleaning routine but also the need for health education among patients. Interestingly, although recent extractions were not significantly correlated with the presence of infections (*p* = 0.301), other dental interventions—such as root canal treatments or recent acute infections—were associated with a doubled risk of oral foci of infection (RR = 2.077; *p* = 0.006), and any dental procedure performed within the last three months increased the relative risk to 2.4 (*p* < 0.001). Also, tooth brushing frequency was an essential indicator: the risk doubled among patients who brushed their teeth only once every few days compared with those with daily hygiene (RR = 2.000; *p* = 0.057), and combining the categories “once a day” and “less frequently” revealed a strong association with the presence of infection (66.0% vs. 35.7%, OR = 3.488). This association highlights the negative impact of inadequate oral hygiene on oral health, especially in a context of increased systemic risk, such as patients scheduled for arthroplasty [34]. Regarding oral hygiene level objectively assessed through the Oral Hygiene Index (OHI), patients with OHI = 2 were more than 14 times more likely to have infectious foci compared to those with OHI < 2, indicating a strong link between oral hygiene score and infection risk (OR = 14.583; *p* < 0.001). Moreover, the presence of any degree of impaired hygiene (OHI ≥ 1) was a robust predictor of oral foci of infection (χ^2^ = 28.868; *p* < 0.001). Although most patients reported daily brushing, only a fraction brushed twice daily, as recommended in current guidelines.

The literature emphasizes that insufficient oral hygiene directly contributes to the development of dental plaque and gingival inflammation, which promote dental caries, gingivitis, periodontitis, tooth loss, halitosis, fungal infections, and other gingival diseases [61]. Tooth brushing frequency is an essential behavioral indicator of oral cavity health. According to the literature, a higher oral hygiene index is frequently associated with inflammatory and infectious risks at gingival and periodontal levels [62]. Inadequate hygiene favors bacterial colonization and persistent chronic inflammation at the periodontal level, which may negatively influence postoperative outcomes in the context of total arthroplasty [63].

Periodontal disease, a chronic inflammatory infectious condition, is one of the most frequent causes of oral infectious foci. The systemic inflammatory process common to these diseases may promote local immune imbalances that contribute to worsening periodontal disease and, implicitly, to maintaining or increasing oral infectious load. Identifying and treating periodontal disease in a timely manner thus becomes essential when planning major orthopedic interventions, especially in patients with chronic inflammatory or degenerative joint pathology. Moreover, multiple studies have demonstrated a bidirectional relationship between chronic periodontal inflammation and rheumatoid arthritis, with both conditions involving common immunological and inflammatory mechanisms [62,63,64,65]. At the same time, patients with advanced hip or knee osteoarthritis are often in a preoperative stage in which untreated dental foci generated by periodontal disease may represent a significant risk for postoperative periprosthetic infections.

To better understand the relationship between oral health and joint disease, a group of researchers conducted a matched case–control analysis focusing on the relationship between periodontitis and a history of osteoarthritis [63]. This study showed that individuals with periodontitis had a significantly higher risk of developing osteoarthritis (OR = 1.15; 95% CI: 1.12–1.17; *p* < 0.001). The link between periodontitis and osteoarthritis was significant in both sexes and across all age groups over 30 years. Women (OR = 1.27) and patients aged over 50 years (OR = 1.21) with periodontitis were particularly prone to developing severe forms of knee or hip osteoarthritis. In addition, patients with periodontitis were more likely to have a history of knee or hip osteoarthritis than those in the control group (OR = 1.11; *p* < 0.001), suggesting that the relationship may also work in the opposite direction. The authors of this study concluded that there is a strong link between periodontitis and knee or hip osteoarthritis—each condition potentially influencing the occurrence of the other—highlighting the importance of an integrated approach to patient care [66]. A research group has pointed out that generalized periodontitis in patients with stage 3 or 4 periodontal disease was associated with significantly higher rates (OR = 2.10) of tooth extractions of periodontal etiology compared to patients in stage 1 and 2, even in compliant patients under supportive periodontal therapy [67].

An OHI score of 2 was found in most patients scheduled for total hip or knee arthroplasty. The significance of the high odds ratio (OR = 16.314) confirms that poor plaque control and calculus deposits have a determinant role in oral bacterial colonization and the presence of oral foci of infection.

This reality reflects not only the lack of a correct cleaning routine but also a possible lack of information or health education. For a patient scheduled to receive a knee or hip joint prosthesis, this can lead to periprosthetic joint infections with serious postoperative complications [68]. With regard to the frequency of dental check-ups, the results indicate a low frequency of routine dental visits, with almost half of the patients reporting that they had not attended a specialist consultation for several years. Non-compliance to regular sessions favors the accumulation of local and systemic risk factors among patients indicated for joint prosthetic treatment [68]. As for the oral symptoms reported at the clinical examination, gingival bleeding, pain, inflammation, or halitosis are not just temporary discomforts—they may indicate deeper conditions, sometimes neglected by patients [69]. The fact that many participants reported such symptoms confirms that oral health status is far from optimal and that there is a need for more prevention and early treatment. The literature highlights that these manifestations should not be overlooked, as they may represent persistent oral foci [70]. In the context of arthroplasty, such sources can represent an additional risk for the occurrence of postoperative infectious complications. All these data outline the profile of the patient scheduled for hip or knee arthroplasty as that of a high-risk patient, who requires more than a simple hygiene recommendation, but a more intense oral disinfection regimen [71,72]. Close collaboration between the orthopedic surgeon and the dentist is not only useful but necessary.

The results obtained through the logistic regression model clearly show that patients with poor oral hygiene, whether they brush their teeth infrequently or present a high OHI score, are significantly more prone to developing oral foci of infection. Poor oral hygiene is not only a local problem but can also have systemic consequences, especially when the patient is about to undergo orthopedic surgery. Regarding brushing habits, it is clear that brushing teeth only once a day is not sufficient in this patient category. The highest risk of oral infectious pathology was determined in patients aged between 50 and 65 years with an OHI score of 2 and tooth brushing once a day or less. In this case, the risk of oral foci of infection becomes extremely high. For these patients, the orthopedic intervention should be postponed until their oral status is stabilized and properly treated [52].

The results of this study invite further consideration as to whether the determinants of oral infection identified in patients scheduled for hip or knee arthroplasty are particular to this group or represent common features across broader surgical or medical populations. Most of detected variables—poor oral hygiene, tooth brushing less often, male sex, and age between 50 and 65 years—are factors that cannot only be referred to orthopedic patients. These are established causes of oral foci of infection irrespective of the systemic condition or operation indication. In patients undergoing elective THA and TKA, the systemic effects of these factors could be more significant because the risks of bacteremia and prosthetic colonization are high. In patients undergoing cardiac valve replacement [73] or organ transplantation [74], the oral hygiene status and bacterial burden are also correlated with postoperative infectious complications. However, the peculiar biological niche of joint prosthesis with low vascularity and scarce immune functions further emphasizes the impact exerted by oral pathogens. In orthopedic surgery, the level of acceptability is very low and even minor oral inflammation is likely to be relevant in a clinical terms because organisms may spread late hematogenously to prosthetic joints [52].

Some limitations of this study need to be considered. First, the single-center design may somewhat restrict the generalization of findings to larger or more diverse samples. Second, given the relatively small sample size, the findings should be viewed as indicative rather than conclusive, and their generalization to larger populations should be made with caution. Third, being a cross-sectional study, associations between oral health factors and the existence of oral foci of infection, but not causality, can be inferred. Fourth, clinical and radiographic examinations were executed by a single examiner, possibly resulting in observer bias despite the self-evident advantage of standardization.

In the context of this study, identifying and eliminating risk factors for oral foci of infection is essential in major orthopedic interventions, such as total hip or knee arthroplasty, since these infections can represent a significant source of hematogenous bacterial dissemination to joint prostheses. Therefore, a multidisciplinary approach that includes rigorous evaluation and treatment of the oral infectious pathology before orthopedic surgery may contribute to the decrease in the incidence of periprosthetic joint infections.

## 5. Conclusions

The comparative analysis of patients scheduled for hip or knee arthroplasty revealed an increased prevalence of oral foci of infection in males, age between 50 and 65 years, tooth brushing once a day or less, and patients with an OHI score of 2. The multivariate analysis shows that patients with a low tooth brushing frequency and high OHI score are much more likely to present oral infectious foci. The risk factors for the presence or oral foci of infection in patients scheduled for total knee or hip arthroplasty require preoperative assessment and management of these factors in order to reduce the risk of the postoperative periprosthetic joint infections.

## Figures and Tables

**Table 1:** Features of subjects with orthopedic diagnosis.

Feature		*n*	%
Sex	Male	29	32.6
	Female	60	67.4
Residence	Urban	61	68.5
	Rural	28	31.5
Age group	50–65 yrs.	28	31.5
	Over 65 yrs.	61	68.5
Smoker	Yes	12	13.5
	No	77	86.5
Chronic alcohol consumption	Yes	16	18.0
	No	73	82.0
Systemic pathology	Yes	50	56.2
	No	39	43.8
OHI score	Score 0	20	22.5
	Score 1	27	30.3
	Score 2	42	47.2
Diabetes mellitus	Yes	38	42.7
	No	51	57.3
Rheumatoid arthritis	Yes	15	16.9
	No	74	83.1
Systemic lupus	Yes	4	4.5
	No	85	95.5
Cancer therapy	Yes	4	4.5
	No	85	95.5
Obesity	Yes	69	77.5
	No	20	22.5
Immunosuppressive therapy	Yes	8	9.0
	No	81	91.0
Orthopedic diagnosis	Knee osteoarthritis	45	50.6
	Hip osteoarthritis	44	49.4
Total		89	100.0

**Table 2:** Prevalence of oral foci of infection.

Oral Foci of Infection	Nr	%
Chronic periapical lesions	12	23.5
Endo-periodontal lesions	21	41.2
Root remnants	43	84.3
Advanced periodontal disease (at least one active PPD ≥ 4 mm)	51	100
Fixed prosthetic with improper marginal fitting	33	64.7

**Table 3:** Pearson Chi-squared test in patients with orthopedic diagnosis—comparisons of study group (OFI) vs. control group (Non-OFI).

Variable		OFI/Non-OFI				
		Presence of Oral Foci of Infection		Absence of Oral Foci of Infection		*p*-Value
		*n*	%	*n*	%	
Sex	Male	21	44.7%	8	19.0%	0.010 *
	Female	26	55.3%	34	81.0%	
Residence	Urban	30	63.8%	31	73.8%	0.311
	Rural	17	36.2%	11	26.2%	
Age group	50–65 yrs.	21	44.7%	7	16.7%	0.004 **
	over 65 yrs.	26	55.3%	35	83.3%	
Smoker	Yes	12	25.5%	-	-	<0.001 **
	No	35	74.5%	42	100.0%	
Chronic alcohol consumption	Yes	8	17.0%	8	19.0%	0.804
	No	39	83.0%	34	81.0%	
Systemic pathology	Yes	24	51.1%	26	61.9%	0.303
	No	23	48.9%	16	38.1%	
Diabetes mellitus	Yes	16	34.0%	22	52.4%	0.081 +
	No	31	66.0%	20	47.6%	
Rheumatoid arthritis	Yes	4	8.5%	11	26.2%	0.026 *
	No	43	91.5%	31	73.8%	
Systemic lupus	Yes	4	8.5%	-	-	0.119
	No	43	91.5%	42	100.0%	
Cancer therapy	Yes	4	8.5%	-	-	0.119
	No	43	91.5%	42	100.0%	
Obesity	Yes	34	72.3%	35	83.3%	0.215
	No	13	27.7%	7	16.7%	
Immunosuppressive medication	Yes	8	17.0%	-	-	0.006 **
	No	39	83.0%	42	100.0%	
Dental check-up	each 12 months	21	44.7%	11	26.2%	0.070 +
	non-compliant	26	55.3%	31	73.8%	
Tooth brushing	once every few days	5	10.6%	-	-	0.057 +
		42	89.4%	42	100.0%	
Tooth brushing	once a day or less	31	66.0%	15	35.7%	0.004 **
		16	34.0%	27	64.3%	
OHI	0	-	-	20	47.6%	<0.001 **
	1	12	25.5%	15	35.7%	
	2	35	74.5%	7	16.7%	
OHI	OHI > = 1	47	100.0%	22	52.4%	<0.001 **
	OHI = 0	-	-	20	47.6%	
OHI	OHI = 2	35	74.5%	7	16.7%	<0.001 **
	OHI < 2	12	25.5%	35	83.3%	
Dental infection in last 3 months	Yes	8	17.0%	-	-	0.006 **
	No	39	83.0%	42	100.0%	
Dental extraction in last 3 months	Yes	8	17.0%	4	9.5%	0.301
	No	39	83.0%	38	90.5%	
Endodontic treatment in last 3 months	Yes	8	17.0%	-	-	0.006 **
	No	39	83.0%	42	100.0%	
Other dental treatments in last 3 months	Yes	17	36.2%	-	-	<0.001 **
	No	30	63.8%	42	100.0%	
Total		47	100.0%	42	100.0%	

* = Statistically significant; ** = Highly statistically significant; + = Marginally significant.

**Table 4:** Risk factors and OR for oral foci of infection (univariate and multivariate analysis).

	Univariate Analysis		Multivariate Analysis		
Risk Factor	OR	95% CI	*p*-Value	OR	95% CI
Male sex	3.433	1.313 ÷ 8.976	0.003 **	12.050	2.363 ÷ 61.446
Age group 50–65 years	4.038	1.494 ÷ 10.918	0.002 **	13.168	2.528 ÷ 68.603
Smoker	2.200	1.723 ÷ 2.810			
Rheumatoid arthritis	0.262	0.076 ÷ 0.901			
Immunosuppressive drug therapy	2.077	1.657 ÷ 2.604			
Tooth brushing once every few days	2.000	1.615 ÷ 2.477			
Tooth brushing once daily or less	3.488	1.457 ÷ 8.351	0.003 **	7.951	2.039 ÷ 31.008
OHI = 2	14.583	5.138 ÷ 41.395	<0.001 **	16.314	4.365 ÷ 60.975
Dental infection in the last 3 months	2.077	1.657 ÷ 2.604			
Root canal treatment in the last 3 months	2.077	1.657 ÷ 2.604			
Other dental treatments in the last 3 months	2.400	1.826 ÷ 3.154			

** = Highly statistically significant.

## Data Availability

The original contributions presented in this study are included in the article. Further inquiries can be directed to the corresponding author.

## Abbreviations

The following abbreviations are used in this manuscript:

**OHI**	Oral Hygiene Index
**OFI**	Oral Foci of Infection
**Md**	Mandibular
**Mx**	Maxillary
**PJI**	Periprosthetic Joint Infections
**THA**	Total Hip Arthroplasty
**TKA**	Total Knee Arthroplasty
